# Extreme Maternal Metabolic Acidosis Leading to Fetal Distress and Emergency Caesarean Section

**DOI:** 10.1155/2013/847942

**Published:** 2013-06-11

**Authors:** Nicolas Cecere, Corinne Hubinont, Arnauld Kabulu Kadingi, Marie-Françoise Vincent, Peter Van den Bergh, Anna Onnela, Philippe Hantson

**Affiliations:** ^1^Department of Intensive Care, Université Catholique de Louvain, Cliniques St-Luc, Avenue Hippocrate 10, 1200 Brussels, Belgium; ^2^Department of Obstetrics, Université Catholique de Louvain, Cliniques St-Luc, 1200 Brussels, Belgium; ^3^Department of Biochemistry, Université Catholique de Louvain, Cliniques St-Luc, 1200 Brussels, Belgium; ^4^Neuromuscular Reference Centre, Université Catholique de Louvain, Cliniques St-Luc, 1200 Brussels, Belgium; ^5^Department of Neonatology, Université Catholique de Louvain, Cliniques St-Luc, 1200 Brussels, Belgium

## Abstract

A 31-year-old pregnant woman (32 + 3 weeks) was admitted with extreme tachypnea. She had a previous history of congenital muscular dystrophy (Ullrich's disease) and isolated glucosuria. The patient had reduced food intake during the last 24 hours prior to admission and vomited twice. Serum glucose level was normal (112 mg/dL), while urinalysis revealed glucosuria 4+ and ketonuria 4+. ABG revealed pH 7.06, PCO_2_ 9 mm Hg, and bicarbonate 2 mmol/L. Anion gap was 28 mmol/L. Tachypnea was a compensatory mechanism for a severe nonlactic metabolic acidosis. The diagnosis of starvation ketoacidosis was established. The patient received supplemental dextrose 10% intravenously and sodium bicarbonate. As fetal heart monitoring was pathological, an emergency caesarean section was performed. Umbilical cord venous pH was 7.01, with PCO_2_ 34 mm Hg and bicarbonate 8 mmol/L. Starvation ketoacidosis is a rare metabolic disorder that may occur mainly in the third trimester of pregnancy. Muscular dystrophy and renal glucosuria were precipitating factors.

## 1. Introduction

Ketoacidosis during pregnancy is a medical emergency for both mother and fetus, often due to uncontrolled diabetes mellitus. We describe a case of extremely severe ketoacidosis with increased anion gap during the third trimester of pregnancy in a nondiabetic woman. The etiology was an unusually short fasting period associated with vomiting (starvation ketoacidosis). Despite the severity of maternal acidosis, the neonatal outcome after an emergency caesarean section was favourable. We discuss the underlying mechanism and the role of other potential contributing factors.

## 2. Case Presentation

**Figure 1:**
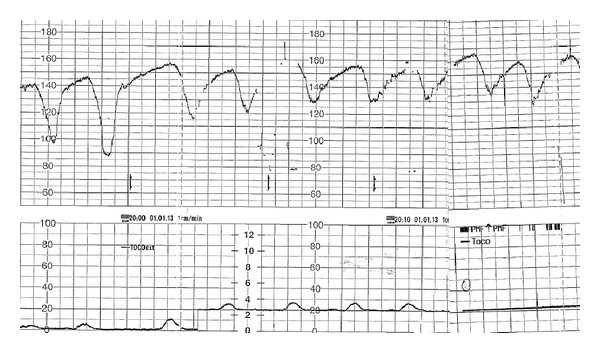
Fetal heart rate monitoring prior to delivery by caesarean section.

A 31-year-old pregnant woman (G2P1A0) was admitted at 32 + 3 weeks of gestation to the intensive care unit (ICU) for acute respiratory distress and acidosis. She had a previous history of congenital muscular dystrophy (Ullrich's disease) and an isolated familial glucosuria due to mutations in the gene encoding for the Na^+^-glucose cotransporter SGLT2. Two years ago, the patient had uneventful pregnancy and delivery. During the current pregnancy, the antenatal care was uneventful until 32 weeks. The patient fell and suffered right external and internal malleolus fractures. For pain relief, she was given a maximal daily dose of 4 grams paracetamol for at least 3 days. She presented at 32 + 3 weeks with nausea and vomiting. Food intake was reduced, but not stopped, only during the last 24 hours prior to admission. Vomiting was limited to two episodes. On arrival, the patient was fully conscious but agitated. Extreme tachypnea (>50/min) was evidenced from physical examination. Temperature was normal (36.4°C). There was no tachycardia. Her arterial blood pressure was 130/70 mm Hg, and peripheral oxygen saturation was 100%. Arterial blood gas measurement at admission revealed severe metabolic acidosis (pH 7.06, PCO_2_ 9 mm Hg, bicarbonate 2 mmol/L, and lactate 0.9 mmol/L (normal value < 1.8)). Anion gap was highly increased (28 mmol/L). Serum glucose was normal (112 mg/dL), creatinine 0.36 mg/dL (NV: 0.50–0.90), and acetone 0.26 g/L. Urinalysis revealed 4+ glucose and 4+ acetone. Paracetamol and salicylates were not detected in the blood. Intramuscular betamethasone for fetal lung maturation was administered before transfer to the university ICU for management and delivery. After 400 mmol of sodium bicarbonate and dextrose 10% infusion (100 mL/hr), there was an improvement of metabolic abnormalities with a rise of arterial pH to 7.52, PCO_2_ to 12 mm Hg, and bicarbonate to 10 mmol/L. Further investigations on the mother's serum and urine collected on admission demonstrated an extremely high serum concentration of 3-hydroxybutyric acid (8020 mmol/L) together with a massive urine excretion (101260 mmol/mol creatinine (NV < 10)). Additionally, there was a mild increase in pyroglutamic aciduria (69 mmol/mol creatinine (NV < 61)). Fetal heart monitoring was highly pathological with repeated complicated decelerations and absence of variability of the fetal heart rate (Figure 1). An emergency caesarean section under general anesthesia was performed 3 hours after ICU admission. An eutrophic female newborn (1890 g) was delivered with an APGAR score of 1 at 1 min and 6 at 5 min. Umbilical cord venous pH was 7.01, with PO_2_ 10.1 mm Hg, PCO_2_ 34 mm Hg, bicarbonate 8 mmol/L, and lactate 7.3 mmol/L. After neonatal resuscitation, the baby was placed on nasal continuous positive airway pressure (CPAP) ventilation and developed moderate respiratory distress. Arterial blood gas analysis at one hour of life showed pH 7.05, PO_2_ 50 mm Hg, PCO_2_ 22 mm Hg, bicarbonate 8 mmol/L, and lactate 12 mmol/L. After evidence of a moderate hyaline membrane disease, mechanical ventilation was started for 4 days, despite the fact that metabolic acidosis was partly compensated by hyperventilation (respiratory rate 65/min). A moderate intraventricular cerebral hemorrhage was noted (grade 2 of Papile), together with periventricular echodensities at serial head ultrasounds.

In the mother, serum bicarbonate concentration stabilized around 20 mmol/L sixteen hours after ICU admission. The patient had an uneventful recovery and was discharged on day 7. One month later, laboratory investigations in nonfasting conditions confirmed isolated glucosuria with normoglycemia and absence of ketonuria. The baby was discharged on day 42, with a normal physical and neurological examination.

## 3. Discussion

Only a few cases of severe starvation ketoacidosis have been reported during late pregnancy [1–5]. The pathophysiology is usually ascribed to a relative insulin resistance, an inadequacy of oral carbohydrate intake, and reduced hepatic glycogen stores [1, 3]. Severe maternal ketoacidosis may have serious consequences on the fetus. In addition to reducing uterine blood flow, increased levels of 3-hydroxybutyrate in the maternal circulation could result in fetal acidosis, reduced PO_2_, and increased lactate levels [6]. Impaired neuronal function or viability may occur.

Our case appears unusual for several reasons. First, ketoacidosis developed in a well-fed pregnant woman, after only a very short period of relative fasting (<24 hours). 

Second, acidosis was much more severe than pH value reported in the literature data. In a recent review on pregnant women presenting ketoacidosis during the last trimester, the extreme arterial pH values ranged from 7.02 to 7.31, with serum bicarbonate ranging between 3.7 and 13.7 mmol/L (average 8.6 mmol/L) [1]. In most of the cases (10/11), however, maternal arterial pH remained above 7.19. Our patient had one of the lowest published bicarbonate and PCO_2_ values despite the very short prodromic period. It is important to mention that the fetal death case report was associated with a very low maternal arterial pH (7.02) [7].

A differential diagnosis with a toxic cause has been considered. Among the medications that may exacerbate starvation ketoacidosis in pregnancy, bêta-agonists used as tocolytics may increase lipolysis, gluconeogenesis, and glycogenolysis [1]. Our patient was not administered bêta-agonists and did not receive high doses of corticosteroids (intramuscular betamethasone) until after presentation which would have exacerbated rather than precipitated the problem. The role of paracetamol toxicity is more complex. Indeed, the differential diagnosis of unexplained metabolic acidosis with increased anion gap should also include pyroglutamic acidemia [8]. Pyroglutamic acid is an intermediate of the *γ*-glutamyl cycle, which is involved in the transport of amino acids across the cell membrane and in the synthesis of glutathione. Acquired pyroglutamic metabolic acidosis was described in adults in association with critical illness, but also in malnourished patients in the context of therapeutic use of paracetamol. The mechanism is unclear but seems to be associated with glutathione deficiency. In the present observation, there was only a slight increase in pyroglutamic acidemia that could certainly not explain the high anion gap metabolic acidosis. 

Third, a possible relationship has to be discussed between starvation ketoacidosis and the preexisting muscular dystrophy and glucosuria. Starvation ketoacidosis was described in two patients who were suffering from the Duchenne muscular dystrophy [9, 10]. However, one patient had a history of type 2 diabetes, and the other had a long-lasting history of dysphagia and malnutrition. Nevertheless, a reduced muscle mass may cause a deficiency of substrate for gluconeogenesis. The role of glucosuria appeared prominent in our patient. It has to be remembered that approximately 180 grams of glucose per day enter the tubular system of the kidneys in a healthy individual with normoglycemia, which is equivalent to approximately one-third of the total energy consumed by the human body. An almost complete renal reabsorption is the rule in healthy adults.

The treatment of starvation ketoacidosis should be mainly based on glucose administration (and insulin if needed). The use of large amounts of sodium bicarbonate may expose to the risk of overshooting metabolic alkalosis, which is a well-known complication of the refeeding syndrome [11].

In conclusion, although familial renal glucosuria may be considered as a benign disorder, it may have precipitated pregnancy-related starvation ketoacidosis in a patient with limited capacity for gluconeogenesis due to congenital muscular dystrophy.
